# TMEM92 drives EMT-associated invasiveness, cisplatin resistance, and immune suppression in head and neck squamous cell carcinoma

**DOI:** 10.1038/s41598-026-49694-7

**Published:** 2026-04-28

**Authors:** Rong He, Yongzhi Wu, Chenzhou Wu, Longjiang Li

**Affiliations:** State Key Laboratory of Oral Diseases, National Clinical Research Center for Oral Diseases, Department of Head and Neck Oncology, West China Hospital of Stomatology, Sichuan University, No. 14, Section 3, Renmin South Road, Wuhou District, Chengdu, Sichuan Province China

**Keywords:** TMEM92, HNSCC, EMT, Cisplatin resistance, Immune suppression, Biomarkers, Cancer, Computational biology and bioinformatics, Immunology, Oncology

## Abstract

Transmembrane protein 92 (TMEM92) has been implicated in tumor progression in several malignancies, yet its role in head and neck squamous cell carcinoma (HNSCC) remains unclear. Here, we systematically investigated the expression pattern, clinical relevance, and biological functions of TMEM92 in HNSCC. Transcriptomic and clinical data from TCGA and GEO were analysed, including bulk, single-cell, and spatial transcriptomic data from GEO. TMEM92 was significantly upregulated in HNSCC, particularly in HPV-negative tumors, and high TMEM92 expression was independently associated with poor overall survival. Single-cell analysis revealed that TMEM92 expression was predominantly enriched in malignant cells. Functional analyses and in vitro experiments demonstrated that TMEM92 promoted cell proliferation, migration, invasion, epithelial–mesenchymal transition (EMT), and cisplatin resistance. Moreover, elevated TMEM92 expression was associated with lymph node metastasis, an immunosuppressive tumor microenvironment, and a poor predicted response to immunotherapy. These findings suggest that TMEM92 plays a critical role in HNSCC progression and may serve as a potential prognostic biomarker and therapeutic target.

**Supplementary Information:**

The online version contains supplementary material available at 10.1038/s41598-026-49694-7.

## Introduction

Head and neck squamous cell carcinoma (HNSCC), the seventh most prevalent malignancy worldwide, arises from the epithelial lining of the mucosal surfaces of the oral cavity, pharynx, and larynx^1^. Despite substantial advances in diagnostic strategies and therapeutic modalities that have improved clinical outcomes, major challenges—including lymph node metastasis, tumor recurrence, and therapeutic resistance—continue to limit the effective management of HNSCC. Nevertheless, 5-year overall survival rates for patients with advanced HNSCC remain as low as 25 to 60%^1,2^. These findings highlight the urgent need to identify reliable diagnostic biomarkers and effective therapeutic targets at an early stage in order to improve patient outcomes and reduce disease burden.

Importantly, HNSCC is a highly heterogeneous disease with marked biological and clinical differences between human papillomavirus (HPV)-positive and HPV-negative tumors. While HPV-positive HNSCC generally exhibits a more favourable response to radiotherapy and systemic treatment, patients with HPV-negative disease often present with more aggressive tumor behaviour, higher rates of locoregional recurrence, and poorer overall survival^1^. In addition, intrinsic and acquired resistance to chemotherapy, particularly cisplatin-based regimens that remain a cornerstone of HNSCC treatment, further contributes to treatment failure^3^. These clinical challenges highlight the importance of identifying molecular determinants associated with tumor progression, therapy resistance, and immune escape, especially in HPV-negative HNSCC subsets.

At the molecular level, HNSCC progression is driven by multiple dysregulated pathways. Epithelial–mesenchymal transition (EMT), a key process underlying tumor invasion and dissemination, promotes the loss of epithelial characteristics and acquisition of mesenchymal features, thereby enhancing migratory and metastatic capacity^4^. Closely related to EMT, focal adhesion signalling regulates cell–extracellular matrix interactions, cytoskeletal remodelling, and mechanotransduction, all of which are essential for cancer cell motility, invasion, and metastatic colonization^5^. Moreover, cisplatin resistance in HNSCC is a multifactorial process involving enhanced DNA damage repair, suppression of apoptosis, cancer stemness, EMT-related plasticity, and remodelling of the tumor microenvironment^6^. Immune evasion is another defining feature of HNSCC, as tumor cells can establish an immunosuppressive microenvironment through altered cytokine signalling, recruitment of suppressive immune cell populations, and upregulation of immune checkpoint pathways, thereby weakening antitumor immunity and limiting therapeutic efficacy^7,8^.

Although several biomarkers have been proposed for HNSCC, including HPV status, EGFR, PD-L1, TP53 alterations, and emerging gene-expression signatures, their clinical utility remains incomplete for accurately predicting metastasis, chemotherapy response, and immune status across diverse patient populations^9–11^. Therefore, the identification of novel biomarkers that integrate tumor-intrinsic malignant behaviour with treatment resistance and immune regulation remains a major unmet need in HNSCC research.

Transmembrane proteins (TMEMs) are integral membrane proteins that span the lipid bilayer and play essential roles in cellular signaling and homeostasis^12^. Emerging evidence suggests that members of the TMEM family contribute to cancer cell dissemination and malignant progression, highlighting their functional importance in tumor biology and their potential as targets for precision oncology^13^. In HNSCC, several TMEM family members—including TMEM16A, TMEM156, TMEM173, and TMEM213—have been identified as novel biomarkers and promising candidates for personalized therapeutic intervention^14^. Given that transmembrane proteins frequently function at the interface of intracellular signalling, cell adhesion, and immune communication, they may play important roles in multiple biological processes involved in HNSCC progression.

To further elucidate the involvement of TMEM proteins in HNSCC, we performed comprehensive bioinformatic analyses and identified TMEM92 as significantly upregulated in HNSCC tissues. Moreover, TMEM92 has been reported to exert tumor-promoting functions in breast carcinoma and pancreatic cancer^15,16^. However, its role in HNSCC has not yet been well characterized. Building upon these findings, the present study systematically investigates the role of TMEM92 in HNSCC malignant progression, with a particular focus on its involvement in tumor growth, metastasis, cisplatin resistance, and immune suppression.

## Results

### TMEM92 is upregulated in HNSCC and exhibits diagnostic potential

**Fig. 1:**
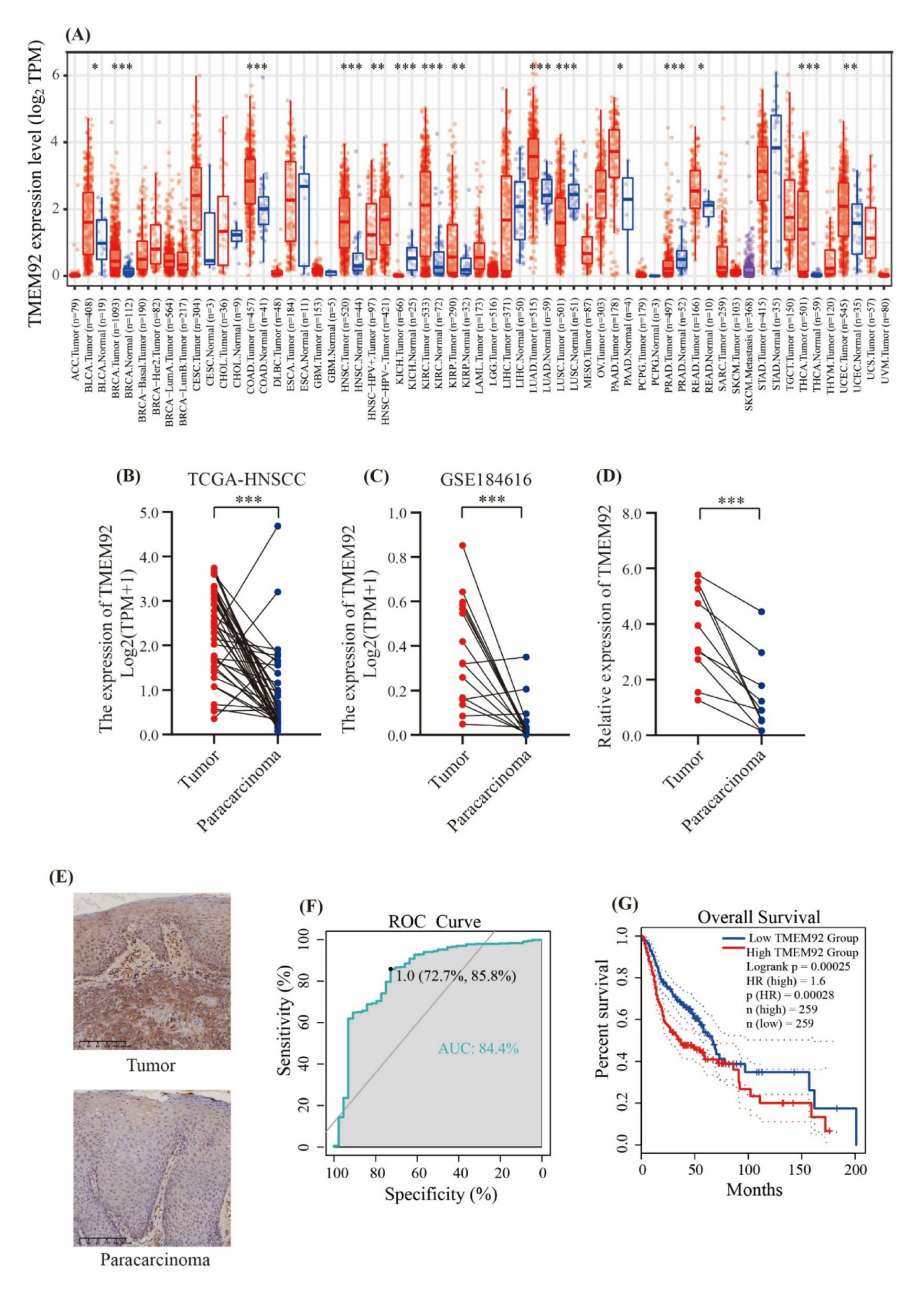
TMEM92 is upregulated in HNSCC and exhibits diagnostic and prognostic value. (**A**) Pan-cancer analysis of TMEM92 mRNA expression levels using the TIMER2.0 database. (**B**) Comparison of TMEM92 expression between HNSCC and matched non-neoplastic tissues from The Cancer Genome Atlas (TCGA) cohort. (**C**) Differential expression of TMEM92 in HNSCC versus normal tissues from the GSE184616 dataset. (**D**) RT-qPCR validation of TMEM92 expression in paired HNSCC and adjacent normal tissues. (**E**) Representative immunohistochemistry (IHC) images showing TMEM92 protein levels in HNSCC and adjacent non-neoplastic tissues. (**F**) Receiver-operating characteristic (ROC) curve analysis evaluating the diagnostic specificity of TMEM92 for HNSCC. (**G**) Kaplan-Meier survival analysis based on TMEM92 expression using data from the GEPIA2.0 database. *P < 0.05; **P < 0.01; ***P < 0.001; ns, not significant.

To systematically evaluate TMEM92 expression across human malignancies, we first analysed data from the TIMER2.0 database. TMEM92 expression was found to be significantly upregulated in the majority of analysed cancer types, including HNSCC, compared with corresponding normal tissues. In contrast, TMEM92 expression was significantly reduced in a limited number of malignancies (e.g., Prostate Adenocarcinoma and Lung Squamous Cell Carcinoma) relative to normal tissues (Fig. 1A). Focusing specifically on HNSCC, TMEM92 expression was significantly higher in HPV-negative tumors than in HPV-positive tumors (Fig. 1A).

To further validate this observation, TMEM92 expression was examined in paired tumor and adjacent normal tissues from two independent cohorts, including 43 matched pairs from the TCGA-HNSCC dataset and 15 matched pairs from the GSE184616 dataset. In both cohorts, TMEM92 expression was markedly elevated in HNSCC tissues compared with the matched normal tissues (Fig. 1B,C). To experimentally confirm these bioinformatic findings, we analysed TMEM92 expression in an independent cohort of 10 paired HNSCC and adjacent non-neoplastic tissue samples. RT-qPCR analysis revealed a significant upregulation of TMEM92 in tumor tissues relative to adjacent normal tissues (Fig. 1D; Supplementary Table S1). In addition, IHC staining of paired samples further demonstrated increased TMEM92 protein expression in HNSCC tissues (Fig. 1E).

Given the marked differential expression of TMEM92 between tumor and normal tissues in HNSCC, we next assessed its diagnostic performance. Receiver operating characteristic (ROC) curve analysis based on TCGA-HNSCC transcriptomic data yielded an area under the curve (AUC) of 0.844, with a sensitivity of 0.727 and a specificity of 0.858, indicating that TMEM92 has potential diagnostic value in HNSCC (Fig. 1F).

### Single-cell RNA-seq analysis reveals tumor-enriched TMEM92 expression in HNSCC

**Fig. 2:**
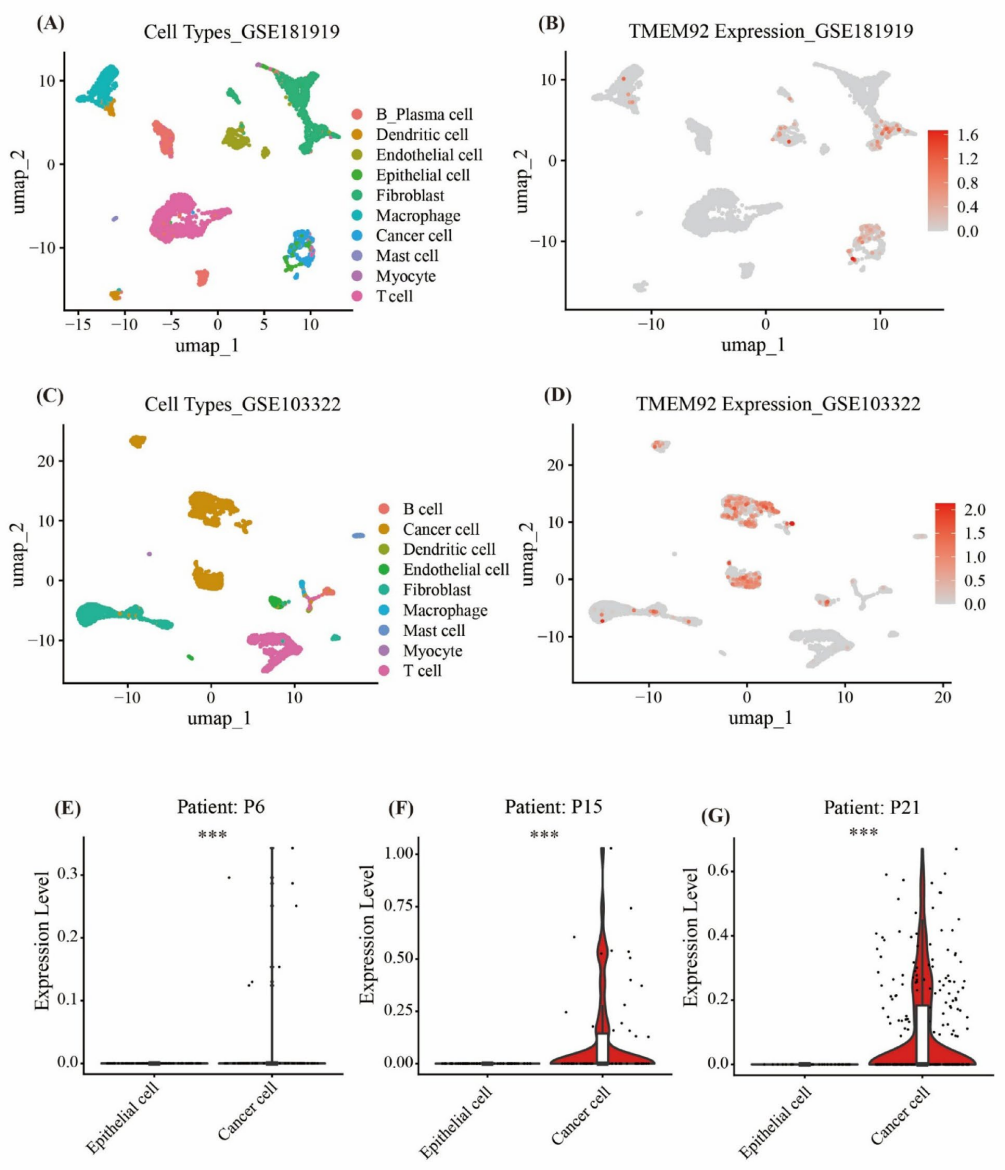
Single-cell RNA-sequencing analysis reveals tumor-enriched expression of TMEM92 in HNSCC. (**A**–**D**) UMAP plots showing the distribution of TMEM92 expression across major cell populations in HNSCC based on single-cell RNA-sequencing datasets GSE181919 and GSE103322. TMEM92 expression was predominantly localized to malignant tumor cells, with detectable expression also observed in epithelial cells, fibroblasts, and endothelial cells. (**E**–**G**) Violin plots comparing TMEM92 expression between malignant tumor cells and epithelial cells in paired single-cell datasets, demonstrating significantly higher TMEM92 expression in HNSCC tumor cells. *P < 0.05; **P < 0.01; ***P < 0.001; ns, not significant.

To further characterize the cellular distribution of TMEM92 within the HNSCC tumor microenvironment, we analysed two single-cell RNA-sequencing datasets, GSE181919 and GSE103322. In both datasets, TMEM92 expression was predominantly localized to HNSCC tumor cells, although detectable expression was also observed in epithelial cells, fibroblasts, and endothelial cells (Fig. 2A–D). Importantly, paired single-cell analyses showed that TMEM92 expression was significantly higher in HNSCC tumor cells than in epithelial cells, supporting a tumor-enriched expression pattern at the single-cell level (Fig. 2E–G). These findings further indicate that the elevated TMEM92 expression observed in bulk HNSCC tissues is largely attributable to tumor cells.

### High TMEM92 expression predicts poor prognosis and advanced tumor stage in HNSCC

To ascertain the clinical relevance of the observed TMEM92 upregulation in HNSCC, we analysed data from the TCGA-HNSCC cohort. An initial survival analysis using GEPIA2 revealed that elevated TMEM92 expression was strongly associated with unfavourable overall survival (OS) (*P* < 0.001), as illustrated in Fig. 1G. We then performed Cox regression analysis to further evaluate its prognostic value. In the univariate model, several factors were significantly linked to patient prognosis, including age, pathologic_T, pathologic_N, and TMEM92 expression itself (*P* < 0.05). Critically, the subsequent multivariate analysis confirmed that TMEM92 expression remained a significant factor, emerging as an independent predictor of prognosis alongside age, pathologic_T, and pathologic_N (*P* < 0.05) (Table 1).

**Table 1:** Univariate and multivariate cox regression analyses of prognostic parameters from the TCGA database.

Parameter	Univariate analysis			Multivariate analysis		
	HR	95% CI	*P*	HR	95% CI	*P*
Age	1.017	1.002–1.031	0.026	1.02	1.004–1.037	0.012
Gender	0.772	0.554–1.074	0.125	0.776	0.549–1.097	0.151
Smoking history	1.017	0.748–1.383	0.913	1.054	0.769–1.446	0.743
Pathologic_T	2.135	1.496–3.048	< 0.001	1.877	1.308–2.694	< 0.001
Pathologic_N	1.871	1.351–2.592	< 0.001	1.837	1.316–2.566	< 0.001
TMEM92	1.254	1.084–1.451	0.002	1.261	1.088–1.461	0.002

Multivariate analysis revealed that Age (*P* < 0.05), pathologic_T (*P* < 0.05), pathologic_N (*P* < 0.05), and TMEM92 expression (*P* < 0.05) independently emerged as independent prognostic factors for HNSCC patients.

**Fig. 3:**
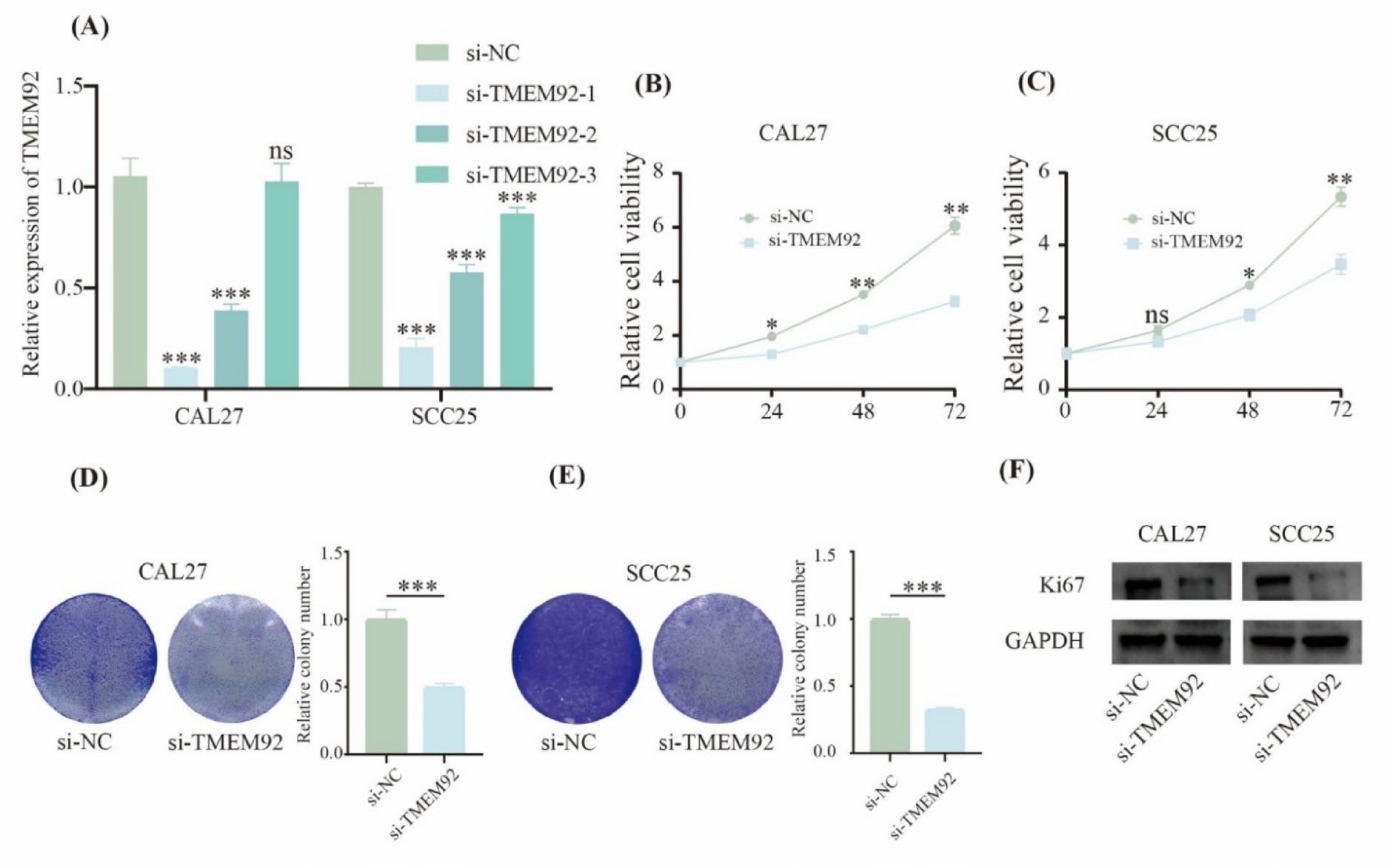
Knockdown of TMEM92 significantly suppresses HNSCC cell proliferation. **(A)** RT-qPCR analysis showing the knockdown efficiency of three siRNAs targeting TMEM92 in HNSCC cells. **(B, C)** CCK-8 experiments demonstrate that si-TMEM92 treatment significantly reduces the viability of HNSCC cells.** (D, E) **Colony formation analysis reveals that TMEM92 knockdown significantly suppresses HNSCC cell proliferation. **(F)** Western blot analysis shows that si-TMEM92 treatment impairs Ki67 expression in HNSCC cells. *P<0.05; **P<0.01; ***P<0.001; ns, not significant.

Focusing on key clinicopathological parameters, a chi-square test revealed that TMEM92 expression positively correlated with tumor size (T stage) (*P* < 0.05) (Table 2). To further investigate the biological impact of TMEM92 in HNSCC, we employed three siRNA targeting TMEM92. Among the designed sequences, siRNA-TMEM92-1 demonstrated the highest knockdown efficiency specifically within the tested CAL27 and SCC25 cell lines. Consequently, it was selected for all subsequent experiments (hereafter referred to as “si-TMEM92”) (Fig. 3A). CCK-8 assays and colony formation demonstrated that silencing TMEM92 significantly impaired HNSCC cell proliferation (Fig. 3B–E). Additionally, Western blot analysis revealed a marked decrease in the proliferation marker Ki67 following TMEM92 knockdown (Fig. 3F)^17^. Collectively, these findings indicate that TMEM92 overexpression is closely associated with poor clinical prognosis, advanced tumor stage, and enhanced proliferative capacity in HNSCC.

**Table 2:** Association of TMEM92 expression with clinicopathological parameters of HNSCC patients.

Characteristics	Total	TMEM92 expression		Χ2	*P*
		Low	High		
Age					
<60	233	121	112	0.569	0.450
≥ 60	286	139	147		
Gender					
Male	383	191	192	0.030	0.862
Female	136	69	67		
Smoking history					
No	222	105	117	1.426	0.232
Yes	283	149	134		
Clinical_T					
T1 + T2	185	104	81	4.380	0.036
T3 + T4	318	148	170		
Clinical_N					
N0	243	125	118	0.341	0.559
N1 + N2+N3	254	124	130		
Clinical_M					
M0	488	248	240	-	0.686
M1	6	4	2		
Clinical_stage					
I+II	117	60	57	0.059	0.810
III+IV	388	194	194		
Pathologic_T					
T1 + T2	183	98	85	2.643	0.104
T3 + T4	273	125	148		
Pathologic_N					
N0	175	93	82	1.473	0.225
N1 + N2+N3	244	115	129		
Pathologic_stage					
I+II	97	48	49	0.035	0.852
III+IV	347	168	179		

The chi-square test indicated that TMEM92 expression was positively correlated with tumor size (T stage). For clinical M, Fisher’s exact test was used because of the small sample size.

### TMEM92 is associated with focal adhesion dynamics and EMT programs in HNSCC

To elucidate the biological pathways associated with TMEM92, we first performed Pearson correlation analyses to identify genes co-expressed with TMEM92 using data from both the TCGA-HNSCC and GSE184616 datasets. Genes were ranked according to the correlation coefficient (R), and the top 500 most strongly correlated genes were subjected to functional enrichment analysis using the DAVID platform. GO analysis revealed that TMEM92 co-expressed genes were significantly enriched in biological processes (BP) related to cell migration, positive regulation of cell migration, and cell adhesion (Supplemental Fig. 1A, B). Consistently, cellular component (CC) analysis showed prominent enrichment in focal adhesion, plasma membrane, cell surface, and membrane organization (Supplemental Fig. 1C, D). Molecular function (MF) analysis further indicated associations with protein binding, integrin binding, and extracellular matrix structural constituent activity (Supplemental Fig. 1E, F). Moreover, KEGG pathway analysis highlighted significant enrichment in focal adhesion, proteoglycans in cancer, regulation of the actin cytoskeleton, and extracellular matrix (ECM)–receptor interaction pathways (Supplemental Fig. 1G, H). Together, these analyses indicate that TMEM92-associated genes are mainly involved in cell–ECM interactions and cytoskeletal organization.

**Fig. 4:**
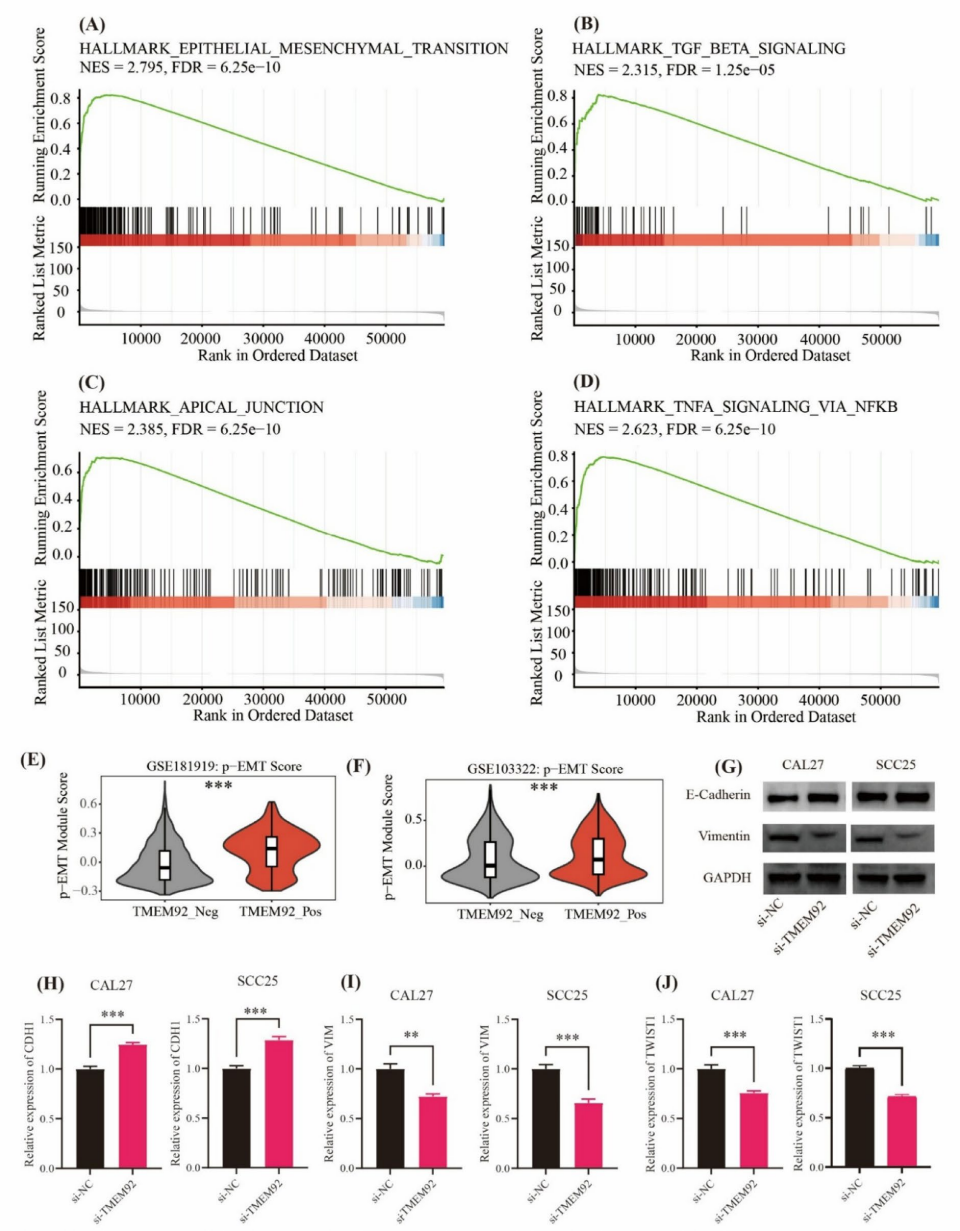
TMEM92 is associated with EMT-related transcriptional programs in HNSCC. **(A–D)** Hallmark gene set enrichment analysis (GSEA) showing significantly enriched pathways in the TMEM92-high group, including EMT **(A)**, TGF-β signaling **(B)**, Apical Junction **(C)**, and TNFA signaling via NFkB** (D)**.**(E–F)** Single-cell RNA-sequencing analysis showing that TMEM92-positive tumor cells exhibit significantly higher partial EMT (p-EMT) scores compared with TMEM92-negative tumor cells. **(G)** Western blot analysis showing that TMEM92 knockdown increased the expression of epithelial marker E-cadherin and decreased the expression of the mesenchymal marker Vimentin. **(H-J)** RT-qPCR showing that TMEM92 knockdown increased the expression of epithelial marker CDH1 and decreased the expression of the mesenchymal marker VIM and TWIST1. *P<0.05; **P<0.01; ***P<0.001; ns, not significant.

To further characterize TMEM92-associated transcriptional programs at the pathway level, Hallmark gene set enrichment analysis (GSEA) was performed. As shown in the bubble plot (Supplemental Fig. 2), the top 10 enriched pathways were predominantly related to cellular plasticity and inflammatory signaling. Specifically, the Epithelial-Mesenchymal Transition and TGF-beta signaling pathways exhibited significant enrichment in the TMEM92-high group (Fig. 4A,B). Notably, the Apical Junction pathway was also positively enriched (Fig. 4C), and enrichment of the TNFA signaling via NF-κB pathway was observed in the TMEM92-high group (Fig. 4D). These findings support an association between TMEM92 and malignant cell state as well as microenvironment-related transcriptional programs at the bulk-transcriptome level.

To validate these transcriptomic findings at the single-cell level, single-cell RNA-sequencing analysis was performed. TMEM92-positive tumor cells displayed significantly higher p-EMT scores than TMEM92-negative tumor cells (Fig. 4E,F). Based on these transcriptomic and single-cell findings, we next performed experimental validation. Western blot analysis demonstrated that TMEM92 knockdown resulted in a marked upregulation of the epithelial marker E-cadherin, accompanied by a concomitant downregulation of the mesenchymal marker Vimentin, consistent with a mesenchymal-to-epithelial transition (MET) phenotype (Fig. 4G)^18,19^. In line with these findings, RT-qPCR analysis further showed that TMEM92 knockdown increased CDH1 expression and decreased VIM and TWIST1 expression (Fig. 4H–J). To further investigate signaling changes associated with TMEM92, we first used the GEPIA2.0 online tool and found that TMEM92 expression was significantly positively correlated with PTK2, TGFB1, and NFKB1 in the TCGA-HNSCC dataset (Supplemental Fig. 3A–C). RT-qPCR analysis after TMEM92 knockdown revealed that PTK2 expression was decreased, whereas TGFB1 showed no significant change and NFKB1 was upregulated (Supplemental Fig. 3D–F). Collectively, these results demonstrate that TMEM92 expression is closely associated with focal adhesion-related pathways and EMT-related transcriptional programs in HNSCC.

### TMEM92 enhances migratory and invasive phenotypes in HNSCC

**Fig. 5:**
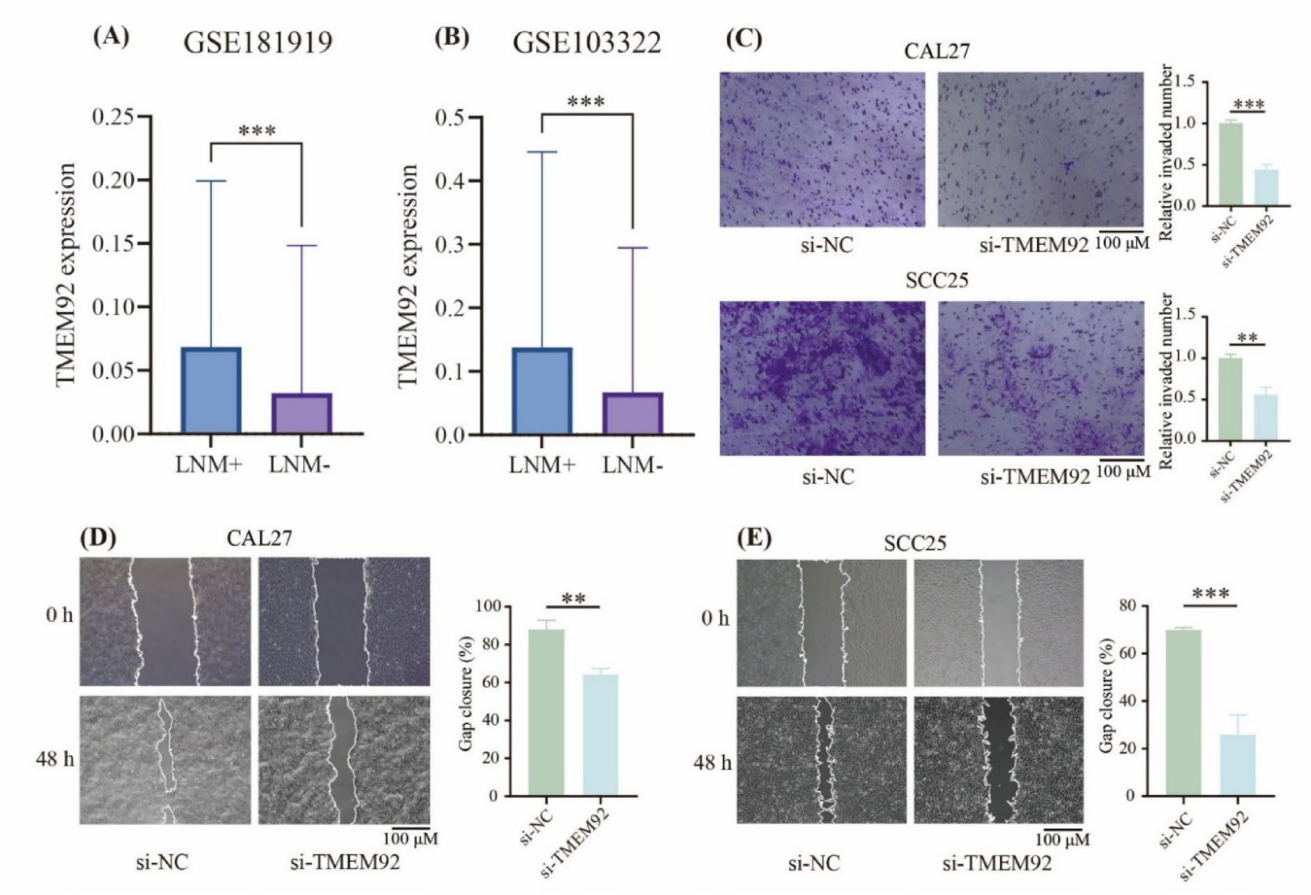
TMEM92 is associated with migratory and invasive phenotypes in HNSCC. **(A–B)** Single-cell RNA-sequencing analysis showing higher TMEM92 expression in primary HNSCC tumor cells from lymph node–positive patients. **(C)** Cell invasion assays demonstrated that si-TMEM92 treatment impaired HNSCC cell invasion. **(D, E)** Wound healing assays demonstrated that si-TMEM92 treatment impaired HNSCC cell migration. LNM+, lymph node metastasis-positive; LNM-, lymph node metastasis-negative. *P<0.05; **P<0.01; ***P<0.001; ns, not significant.

In the TCGA-HNSCC dataset, no significant association was observed between TMEM92 expression and lymph node status. However, given that focal adhesion dynamics and EMT are well-established drivers of lymph node metastasis, we sought to further investigate the potential involvement of TMEM92 in HNSCC dissemination. Notably, single-cell RNA-sequencing analyses revealed that TMEM92 expression in primary HNSCC tumor cells from patients with lymph node metastasis was markedly higher than that in primary tumor cells from lymph node–metastasis–free patients (Fig. 5A,B). To experimentally validate the functional contribution of TMEM92 to HNSCC progression, we next performed transwell invasion and wound-healing assays. Silencing TMEM92 expression resulted in a significant reduction in both the migratory and invasive capacities of HNSCC cells (Fig. 5C–E). Collectively, these findings indicate that TMEM92 promotes HNSCC cell motility and invasiveness, thereby potentially contributing to tumor progression and metastatic potential.

### TMEM92 contributes to cisplatin resistance in HNSCC

**Fig. 6:**
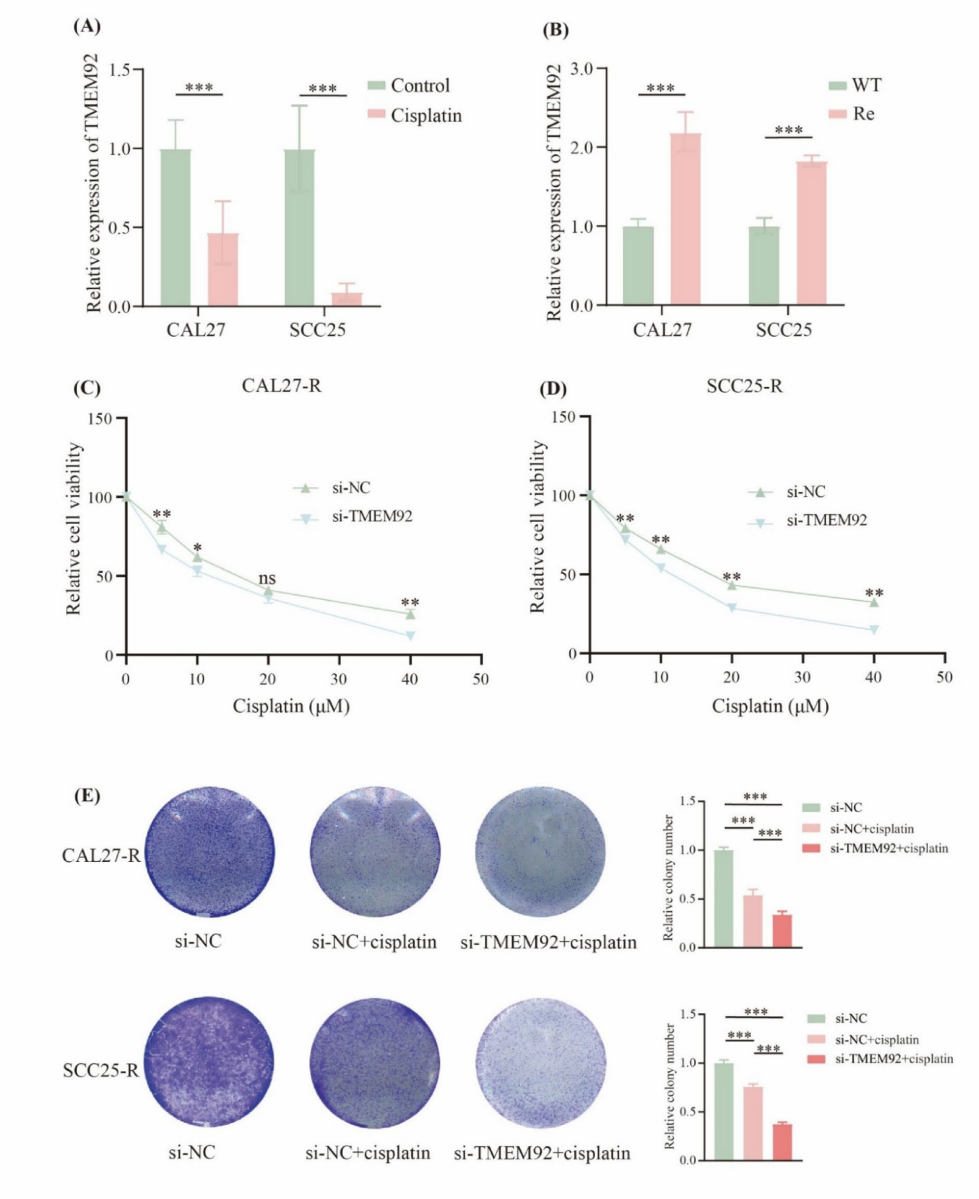
Downregulation of TMEM92 enhances the sensitivity of HNSCC cells to cisplatin. **(A)** Cisplatin treatment (10 μM, 24 hours) downregulated TMEM92 expression in HNSCC cells. **(B)** TMEM92 was significantly increased in HNSCC-R cells. **(C, D)** CCK-8 experiment showed that cisplatin treatment (48 hours) impaired HNSCC cell viability, and that co-treatment with si-TMEM92 further enhanced this effect. **(E)** Colony formation experiments showed that cisplatin treatment (10 μM, 7 days) inhibited cell proliferation and treatment with si-TMEM92 further suppressed cell proliferation. WT, wild type; Re, resistance. *P<0.05; **P<0.01; ***P<0.001; ns, not significant.

Cisplatin-based concurrent chemoradiotherapy remains the gold standard for treating locally advanced HNSCC^20^. However, prolonged cisplatin treatment often leads to the development of chemoresistance, resulting in treatment failure and diminished prognosis^21^. Overcoming cisplatin resistance is therefore a major challenge in HNSCC, and sensitizing tumors to chemotherapy is crucial for improving clinical outcomes. Dose–response curve analysis after 48h of cisplatin treatment confirmed the successful establishment of cisplatin-resistant HNSCC cell lines, as reflected by markedly higher IC50 values in resistant cells than in their parental counterparts. Specifically, the IC50 values were 34.76 µM and 31.05 µM in CAL27-R and SCC25-R cells, respectively, compared with 9.46 µM in CAL27 cells and 6.09 µM in SCC25 cells (Supplemental Fig. 4A, B). Interestingly, RT-qPCR analysis revealed that exposure to cisplatin significantly decreased TMEM92 expression (Fig. 6A). More importantly, TMEM92 expression was significantly increased in cisplatin-resistant HNSCC cells (Fig. 6B). To determine whether TMEM92 could serve as a therapeutic target for overcoming drug resistance, we performed functional assays. CCK-8 assays showed that TMEM92 silencing significantly reduced the viability of both parental and resistant HNSCC cells treated with various concentrations of cisplatin (Fig. 6C,D, Supplemental Fig. 5A, B). Furthermore, colony formation assays showed that while cisplatin treatment alone inhibited cell proliferation, the combination of cisplatin treatment with TMEM92 silencing led to a more pronounced suppression of proliferation (Fig. 6E). Together, these findings suggest that silencing TMEM92 enhances cisplatin sensitivity and may represent a promising therapeutic strategy for overcoming chemoresistance in HNSCC.

### TMEM92 is associated with an immunosuppressive microenvironment in HNSCC

Hallmark GSEA revealed that multiple immune- and inflammation-related pathways were significantly enriched in the TMEM92-high group, including Interferon alpha response, Interferon gamma response, TNFA signaling via NFkB, Inflammatory response, Complement, and TGFβ signaling (Supplemental Fig. 2)^22–24^. Based on these transcriptomic findings, we further investigated the association between TMEM92 expression and immune cell infiltration in HNSCC using the TIMER2.0 database. TMEM92 expression was negatively correlated with tumor purity, indicating a potential association with the tumor immune microenvironment. Among specific immune cell populations, TMEM92 expression showed positive correlations with the infiltration of CD8⁺ T cells (Rho = 0.304, *P* = 5.29 × 10^−12^) and myeloid dendritic cells (Rho = 0.327, *P* = 1.04 × 10^−13^), while weaker or non-significant correlations were observed with B cells, macrophages, and neutrophils (Supplemental Fig. 6A).

To further characterize immune cell composition, single-sample gene set enrichment analysis (ssGSEA) was performed to evaluate the associations between TMEM92 expression and the relative abundance of 28 immune cell subsets in HNSCC (Supplemental Fig. 6B). High TMEM92 expression was associated with increased proportions of central memory CD8⁺ T cells, central memory CD4⁺ T cells, natural killer T cells, regulatory T cells, γδ T cells, CD56^bright natural killer cells, activated dendritic cells, and monocytes. Conversely, TMEM92 expression was negatively associated with the abundance of activated CD8⁺ T cells, activated CD4⁺ T cells, mast cells, eosinophils, T follicular helper cells, immature B cells, and activated B cells. Notably, TIMER2.0 and ssGSEA capture immune infiltration from different analytical perspectives, with TIMER2.0 reflecting overall immune cell abundance and ssGSEA providing insight into functional immune cell subsets. In addition, spatial transcriptomic analysis of the GSE208253 dataset revealed that increasing distance from TMEM92-positive spots was significantly associated with higher overall T-cell abundance estimated by deconvolution (Spearman Rho = 0.319, *P* < 0.001), and also showed a significant positive correlation with CD8⁺ cytotoxic T-cell activity, as assessed by module score (Spearman Rho = 0.104, *P* < 0.001) (Supplemental Fig. 7A, B).

Subsequently, we investigated the association between TMEM92 expression and immune-related molecular signatures using the TISIDB database (Supplemental Fig. 8A). We found that TMEM92 expression was significantly positively correlated with multiple immunoinhibitors in HNSCC, including PD-L2 (Rho = 0.295), PVRL2 (Rho = 0.219), TGFB1 (Rho = 0.434), and TGFBR1 (Rho = 0.209). In contrast, TMEM92 expression was negatively correlated with several immunostimulators, including TNFRSF13B (Rho = − 0.238), TNFRSF17 (Rho = − 0.254), TNFRSF18 (Rho = − 0.218), TNFSF18 (Rho = − 0.253), TNFRSF13C (Rho = − 0.369), CD40LG (Rho = − 0.220), CXCR4 (Rho = − 0.208), and HHLA2 (Rho = − 0.222). Positive correlations were also observed with several immune regulatory molecules, including NT5E (Rho = 0.438), CD276 (Rho = 0.426), and PVR (Rho = 0.466). Additionally, TMEM92 expression was positively correlated with MHC-related genes, including B2M (Rho = 0.284), HLA-A (Rho = 0.285), HLA-B (Rho = 0.316), HLA-C (Rho = 0.307), HLA-E (Rho = 0.314), HLA-F (Rho = 0.319), TAP1 (Rho = 0.218), TAP2 (Rho = 0.262), and TAPBP (Rho = 0.227). Finally, analysis of TCGA-HNSCC data revealed that patients with high TMEM92 expression exhibited significantly higher TIDE scores, indicative of a less favourable predicted response to immunotherapy (Supplemental Fig. 8B). Consistently, the high-expression group displayed increased T-cell exclusion scores and reduced responder scores compared with the low-expression group.

## Discussion

TMEMs have emerged as important regulators of tumor initiation and progression, with dysregulated expression patterns reported across multiple cancer types^12^. Depending on cellular context, TMEM family members may function as oncogenes or tumor suppressors. Although TMEM92 has recently been identified as a prognostic biomarker in breast and pancreatic cancers^15,16^, its expression pattern, biological function, and clinical relevance in HNSCC have remained largely unexplored. In the present study, we identified TMEM92 as a clinically relevant molecule in HNSCC and found that its upregulation was associated with aggressive clinicopathological features, cisplatin resistance, and an immunosuppressive tumor microenvironment. Rather than serving as an isolated marker, TMEM92 appears to be linked to several biological processes that are central to HNSCC progression.

Our integrative analyses revealed that TMEM92 expression was significantly elevated in HNSCC compared with adjacent normal tissues across multiple independent cohorts and was particularly enriched in HPV-negative tumors. Although the underlying mechanism was not directly investigated in the present study, this finding may reflect the distinct biological and clinical features of HPV-negative HNSCC, which is more frequently associated with TP53 mutation, greater genomic instability, and exposure-related carcinogenesis^1^. These characteristics are often linked to more aggressive disease behaviour and poorer clinical outcomes. Accordingly, the preferential upregulation of TMEM92 in HPV-negative tumors suggests that it may be associated with molecular programs that are more active in this subgroup. In addition, the tumor cell–predominant expression pattern observed in single-cell analyses, together with its association with unfavourable survival, higher T stage, and independent prognostic significance, supports the potential clinical relevance of TMEM92 in HNSCC. Although its diagnostic performance alone may not be sufficient for standalone clinical use, the discriminatory ability observed in our analysis suggests that TMEM92 may still have value when interpreted alongside established clinicopathological or molecular indicators.

Functionally, our data support a role for TMEM92 in promoting malignant progression in HNSCC. TMEM92 knockdown consistently impaired cell proliferation, colony formation, migration, and invasion, indicating that it contributes to both tumor growth and metastatic phenotypes. Importantly, these effects were supported not only by functional assays but also by enrichment analyses linking TMEM92 to focal adhesion-related pathways, cytoskeletal organization, and migratory programs. Mechanistically, TMEM92 was closely associated with EMT-related transcriptional features, and its silencing shifted cells toward a more epithelial phenotype. Since EMT and focal adhesion remodelling are both central to tumor dissemination and cellular plasticity in HNSCC, our findings suggest that TMEM92 may facilitate invasion at least in part through coordination of these processes^19^. To further explore the molecular pathways associated with TMEM92, we used the GEPIA2.0 online tool and found that TMEM92 expression was significantly positively correlated with PTK2, TGFB1, and NFKB1 in the TCGA-HNSCC dataset. Following TMEM92 knockdown, RT-qPCR analysis showed that PTK2 expression was significantly decreased, whereas TGFB1 showed no obvious change and NFKB1 was upregulated. These findings suggest that the link between TMEM92 and focal adhesion signaling may be more direct, as reflected by the reduction in PTK2 expression after TMEM92 silencing. By contrast, the absence of a clear change in TGFB1 expression may indicate that the association between TMEM92 and TGF-β signaling is indirect, context-dependent, or not primarily regulated at the transcriptional level^24,25^. Similarly, the upregulation of NFKB1 after TMEM92 knockdown may reflect activation of a stress-responsive or compensatory feedback program caused by signaling rewiring, rather than a straightforward downstream effect of TMEM92^26,27^. Interestingly, although bulk transcriptomic data did not show a clear association between TMEM92 and lymph node status, single-cell analyses indicated higher TMEM92 expression in primary tumor cells from patients with lymph node metastasis. This discrepancy likely reflects the limitation of bulk tissue profiling in capturing tumor cell–intrinsic metastatic features and highlights the value of single-cell approaches in refining the interpretation of HNSCC heterogeneity. Therefore, while these data support an association between TMEM92 and focal adhesion/EMT-related programs, the precise relationship of TMEM92 with TGF-β and NF-κB signaling requires further mechanistic investigation.

Another important aspect of this study is the potential involvement of TMEM92 in therapeutic resistance. Cisplatin-based chemoradiotherapy remains a cornerstone of treatment for locally advanced HNSCC, yet resistance continues to limit its efficacy^21^. Our results showed that TMEM92 was elevated in cisplatin-resistant HNSCC cells, and that its silencing enhanced cisplatin sensitivity, suggesting that TMEM92 may contribute to resistance-related cellular adaptation. Notably, the sensitizing effect of TMEM92 knockdown was observed in both parental and resistant cell contexts, supporting a broader role for TMEM92 in modulating cisplatin response rather than merely reflecting the resistant state itself. Taken together, these findings suggest that TMEM92 may represent a potentially actionable target for improving cisplatin efficacy in HNSCC.

In parallel, immune analyses indicated that high TMEM92 expression was associated with a less favourable immune contexture, including reduced effector T-cell activity, enrichment of immunoregulatory populations, and increased expression of inhibitory immune signalling molecules. Although bulk immune deconvolution suggested a positive association between TMEM92 expression and CD8⁺ T-cell infiltration, spatial transcriptomic analysis indicated that both overall T-cell abundance and CD8⁺ cytotoxic T-cell activity increased with greater distance from TMEM92-positive regions. This apparent discrepancy suggests that TMEM92-high tumors may retain immune cell infiltration at the whole-tumor level, whereas TMEM92-positive niches exhibit relative spatial separation from effector T cells, consistent with ineffective antitumor immune engagement rather than productive cytotoxic immunity. The positive association with TIDE score and T-cell exclusion further suggests that TMEM92-high tumors may be less responsive to immunotherapy. Taken together, these observations raise the possibility that TMEM92 is involved not only in tumor-intrinsic aggressiveness, but also in shaping a microenvironment that favours therapeutic failure. Because these observations were primarily derived from computational immune deconvolution and experimental models, their precise biological basis still requires further clarification. Nonetheless, they support the view that TMEM92 may have broader relevance than a conventional prognostic marker alone.

Several limitations should also be acknowledged. First, although our findings suggest links between TMEM92 and EMT, cisplatin resistance, and immune suppression, these associations have not yet been directly validated in clinical HNSCC specimens. Future studies using well-annotated clinical cohorts, including tissue microarray-based analyses combined with multiplex immunohistochemistry, will be needed to further confirm these findings. Second, the biological basis for the enrichment of TMEM92 in HPV-negative tumors remains speculative at present. Additional mechanistic studies will be needed to determine whether TMEM92 is functionally linked to molecular drivers that are more characteristic of HPV-negative HNSCC, such as mutant p53-associated pathways. Third, although our supplementary pathway analyses provide initial clues regarding PTK2/FAK-, TGF-β-, and NF-κB-related signaling, these observations remain exploratory and require further validation by dedicated mechanistic experiments, including rescue studies and pathway activity assays.

Notwithstanding these limitations, the present study identifies TMEM92 as a previously underappreciated molecule associated with HNSCC progression. Our data support its relevance to aggressive clinicopathological features, poor prognosis, cisplatin resistance, and immune suppression. Overall, these findings provide a rationale for further investigation of TMEM92 as a potential biomarker and therapeutic target in HNSCC, while also underscoring the need for additional clinical and mechanistic validation.

## Methods

### Bulk transcriptomic and clinical data acquisition

For this study, transcriptomic expression profiles and corresponding clinical information for HNSCC were obtained from The Cancer Genome Atlas (TCGA) and the Gene Expression Omnibus (GEO) databases. The TCGA-HNSCC cohort included RNA-sequencing data from 520 primary tumor samples and 44 tumor-adjacent normal tissues. Notably, 43 of these normal tissues were patient-matched to their corresponding tumors; these specific pairs were selected for the paired differential expression analysis. An independent GEO cohort (GSE184616), comprising 15 paired tumor and adjacent normal samples, was used for external validation.

Clinical parameters for TCGA-HNSCC patients, including age, gender, overall survival, survival status, and American Joint Committee on Cancer (AJCC) TNM staging, were retrieved from the TCGA portal. All datasets were downloaded in standardized formats and processed using R software for subsequent analyses^28^.

### Single-cell transcriptomic analysis and p-EMT scoring

Publicly available single-cell RNA-sequencing datasets for HNSCC (GSE181919 and GSE103322) were obtained from the GEO database^29,30^. Pre-filtered gene expression matrices and corresponding cell-type annotations provided by the original studies were directly used for downstream analyses. Data processing and visualization were performed using the Seurat (version 5.0) R package. Gene expression values were log-normalized with a scale factor of 10,000. Uniform Manifold Approximation and Projection (UMAP) was employed to visualize the distribution of TMEM92 expression across major cellular populations using the original dimensional reduction coordinates. Differential expression of TMEM92 was assessed using the Wilcoxon rank-sum test across different cellular and clinical subgroups. Malignant cells were divided into TMEM92-positive and TMEM92-negative groups based on expression counts (> 0). Partial EMT (p-EMT) scores were calculated using the AddModuleScore function in Seurat according to the gene set defined by Puram et al. and were compared between the two groups^30^.

### GO and KEGG functional enrichment analysis

An analysis was conducted on the 500 genes most strongly correlated with TMEM92, sourced from the TCGA-HNSCC and GSE184616 datasets, using the Database for Annotation, Visualization, and Integrated Discovery (DAVID) platform. The species for this investigation was set to Homo sapiens, and official gene symbols were utilized for identification. This procedure yielded enrichment data for both Gene Ontology (GO) functional annotations and Kyoto Encyclopedia of Genes and Genomes (KEGG) pathways^31,32^. The top six leading results were selected for presentation and further analysis.

### Gene set enrichment analysis (GSEA)

To explore the biological pathways associated with TMEM92 expression, Gene Set Enrichment Analysis (GSEA) was performed using the TCGA-HNSCC cohort. RNA-seq expression data (TPM normalized) were log2-transformed, and patients were stratified into TMEM92-High and TMEM92-Low groups based on the median expression level. Differential expression analysis was conducted using the limma R package to calculate the log_2_ fold change (logFC) and *P*-values for all genes. A ranked gene list was generated based on the metric sign(logFC)×(− log10(*P*-value)). GSEA was implemented via the clusterProfiler R package against the Hallmark gene sets (MSigDB v2025.1), with an adjusted *P*-value < 0.05 considered statistically significant^33^.

### Spatial transcriptomic analysis

The HNSCC spatial transcriptomic dataset GSE208253 was obtained from GEO and processed using Seurat (v4.3.0). Data normalization and variance stabilization were performed using SCTransform. Spots with detectable TMEM92 expression were defined as TMEM92-positive (TMEM92+) spots, and the Euclidean distance from each spot to the nearest TMEM92 + spot was calculated based on spatial coordinates.

For spot annotation and immune cell estimation, a matched HNSCC single-cell RNA-sequencing dataset (GSE103322) was used as the reference. Cell type transfer and deconvolution were performed using FindTransferAnchors and TransferData in Seurat. A CD8⁺ cytotoxic T-cell activity score was calculated using AddModuleScore based on the gene set CD8A, CD8B, NKG7, PRF1, and GZMB. Spearman’s rank correlation analysis was used to evaluate the associations between the distance to TMEM92 + spots and immune-related features, including overall T-cell abundance and CD8⁺ cytotoxic activity.

### Cell culture and establishment of cisplatin-resistant cell lines

The human HNSCC cell lines CAL27 and SCC25 were obtained from Procell Life Sciences and Technologies (Wuhan, China), and both were identified by short tandem repeat sequence analysis at Genecopoeia (Guangzhou, China). The HNSCC cell lines, CAL27 and SCC25, were cultivated in DMEM and DMEM/F12 media, respectively, with each formulation containing 10% FBS. These cultures were maintained within a humidified incubator at 37 °C in a 5% CO_2_ atmosphere. To induce drug resistance, cisplatin-resistant HNSCC populations were established through gradual exposure to escalating doses of cisplatin^34^. The initial phase of this protocol involved growing the CAL27 and SCC25 lines in a complete medium containing 1 µM cisplatin, with the medium being replaced every three days to ensure a stable drug level. Subsequently, the cisplatin concentration was gradually increased to 2 µM, 4 µM, 6 µM, and 10 µM. The cisplatin concentration was appropriately reduced if excessive cell death was observed during this process. The establishment of cisplatin-resistant cell lines was terminated when cells could divide normally in medium containing 10 µM cisplatin. After two months of induction, the cell status stabilized, and they continued to proliferate and passage. The induced drug-resistant cell lines were named CAL27-R and SCC25-R (collectively referred to as HNSCC-R) for subsequent experiments. To maintain cisplatin resistance, medium containing 10 µM cisplatin was used every 7 days. All methods were performed in accordance with the relevant guidelines and regulations.

### RNA isolation and RT-qPCR

The isolation of total RNA was accomplished using a TRIzol-based reagent (Servicebio) while adhering to the manufacturer’s supplied protocol. Subsequently, this RNA was converted into cDNA via reverse transcription employing the Revertaid First Strand cDNA Synthesis Kit (Thermo Scientific). Quantitative PCR analyses were conducted with the 2x SYBR Green qPCR Master Mix (Low ROX, Bimake.com) in accordance with the provided guidelines. Relative gene expression levels were calculated using the 2^−ΔΔCt^ formula, for which GAPDH served as the endogenous normalization control. The synthesis of all primers used in this study was carried out by Sangon Biotech (Shanghai, China), and the specific sequences are provided below: GAPDH forward, 5′-GGAGCGAGATCCCTCCAAAAT-3′, and reverse, 5′-GGCTGTTGTCATACTTCTCATGG-3′; TMEM92 forward, 5′-CATCCTGTCCGTCTTTTGCAT-3′, and reverse, 5′-GAAAGGGATACTCTGACCCTCT-3′; CDH1 forward, 5′-ATTTTTCCCTCGACACCCGAT-3′, and reverse, 5′-TCCCAGGCGTAGACCAAGA-3′; VIM forward, 5′-GACGCCATCAACACCGAGTT-3′, and reverse, 5′-CTTTGTCGTTGGTTAGCTGGT-3′; TWIST1 forward, 5′- GTCCGCAGTCTTACGAGGAG-3′, and reverse, 5′-GCTTGAGGGTCTGAATCTTGCT-3′; PTK2 forward, 5′-TGGTGCAATGGAGCGAGTATT-3′, and reverse, 5′- CAGTGAACCTCCTCTGACCG-3′; TGFB1 forward, 5′-GGCCAGATCCTGTCCAAGC-3′, and reverse, 5′-GTGGGTTTCCACCATTAGCAC-3′; NFKB1 forward, 5′- AACAGAGAGGATTTCGTTTCCG-3′, and reverse, 5′-TTTGACCTGAGGGTAAGACTTCT-3′.

### siRNA-mediated TMEM92 knockdown

For experiments requiring gene knockdown, small interfering RNA (siRNA) designed to target TMEM92 were procured from Sangon Biotech (Shanghai, China). Introduction of these molecules into HNSCC cells was achieved through transient transfection with the Lipofectamine 3000 reagent (Invitrogen), adhering to the manufacturer’s specified protocol. The specific oligonucleotide sequences are detailed below:

**Table:** 

Target	Sense (5′-3′)	Antisense (5′-3′)
NC	UUCUCCGAACGUGUCACGUTT	ACGUGACACGUUCGGAGAATT
si-1	CCAGAGAGGGUCAGAGUAUUU	AUACUCUGACCCUCUCUGGUU
si-2	CCAAAAGAUUGCAGCCAAAUU	UUUGGCUGCAAUCUUUUGGUU
si-3	GCAUCUGUGGCCUGGCUAAUU	UUAGCCAGGCCACAGAUGCUU

### Immunohistochemistry

Immunohistochemistry was performed on 4-µm sections from formalin-fixed, paraffin-embedded (FFPE) HNSCC blocks obtained from West China Stomatological Hospital. Mounted sections were first deparaffinized, rehydrated, and underwent microwave-based antigen retrieval in citrate buffer. After blocking endogenous peroxidase and non-specific binding sites, the slides were probed overnight at 4 °C with an anti-TMEM92 primary antibody (Novus Biologicals, NBP2-38013, 1:100). Detection was accomplished using a biotinylated secondary antibody (Abcam, ab6721, 1:500) and a streptavidin-HRP conjugate, with DAB as the chromogen. Finally, sections were counterstained with hematoxylin, dehydrated, mounted, and digitally scanned at 10x magnification (KF-PRO-005-EX scanner). All methods were performed in accordance with the relevant guidelines and regulations and the Declaration of Helsinki.

### Western blot assay

For protein extraction, cells were lysed on ice using an ice-cold RIPA buffer (Beyotime, China) supplemented with a protease inhibitor cocktail (Roche, Switzerland). Protein concentration was determined via the Bradford Protein Assay (Bio-Rad Laboratories, USA). Following denaturation at 100 °C for 10 min, protein samples underwent separation by SDS-PAGE. To optimize transfer efficiency for proteins of varying molecular weights, the gels were carefully partitioned into horizontal sections based on the pre-stained protein standards prior to the transfer process. These gel sections were then transferred to PVDF membranes under optimized conditions specific to their respective molecular weights. The resulting membrane strips were then probed with primary antibodies for GAPDH (proteintech, 60004-1-Ig, 1:10,000), Ki67 (Abmart, TW0001, 1:500), E-Cadherin (HUABIO, ET1607-75, 1:1000), and Vimentin (Cell Signaling Technology, 5741, 1:500). After incubation with appropriate HRP-conjugated secondary antibodies, bands were visualized using a ChemiDoc imaging system. To ensure full transparency and compliance with the journal’s digital image and integrity policies, original images of all membrane strips from three independent biological replicates, including visible membrane edges, are provided in the Supplementary Information file.

### Cell proliferation assay

To assess cell viability, cells were first plated in triplicate within 96-well plates, with each well containing 5 × 10³ cells in a 100 µL volume. Upon completion of the designated treatment period, 10 µL of CCK-8 reagent (biosharp, China) was introduced into every well. The plates were subsequently incubated for 2 h in a humidified environment at 37 °C with a 5% CO_2_ atmosphere. Finally, the optical density was quantified at a wavelength of 450 nm utilizing a Spectramax Microplate^®^ Spectrophotometer (Molecular Devices LLC, Sunnyvale, CA, USA).

### Colony formation assay

To assess colony formation, HNSCC cells were plated in 6-well plates at a concentration of 2000 cells per well and allowed to grow for 10 days. Following this incubation period, the resulting cell colonies, defined as clusters containing over 50 cells, were fixed using 4% paraformaldehyde (Biosharp, BL539A). The fixed colonies were subsequently rendered visible with a crystal violet stain (Beyotime, C0121), and the total number in each well was then enumerated under an optical microscope.

### Transwell invasion assay

A transwell assay was employed to determine the invasive potential of the cells. For this procedure, the membranes of 24-well culture inserts containing 8-µm pores (BD Biosciences, USA) were coated with Matrigel (1 mg/ml; BD Biosciences). These coated inserts were then incubated for 6 h at 37 °C to allow the Matrigel to solidify. Following this preparation, HNSCC cells that had been treated with si-TMEM92 were seeded into the upper chambers, which contained serum-free medium to minimize the influence of cell proliferation during the assay period. A volume of 0.6 ml complete medium, serving as a chemoattractant, was added to the lower chamber. Because CAL27 and SCC25 cells exhibit relatively moderate baseline motility, a 72-hour incubation period under serum-free conditions was used to allow sufficient invasion and to maximize the observable phenotypic differences between groups. After 72 h, cells were washed twice with PBS, fixed with paraformaldehyde, and stained with Giemsa Stain solution (Solarbio Inc, Beijing, China). To isolate the invasive population, any cells remaining within the upper chamber were gently removed with a cotton swab. Subsequently, the membranes were photographed to document the cells that had successfully migrated through to the bottom surface. The number of invaded cells was quantified using ImageJ software.

### Wound-healing migration assay

To evaluate cellular migration, a wound healing assay was performed. First, cells were cultured in 6-well plates until the cell layer reached approximately 90% confluence. A uniform, linear scratch was then introduced into the monolayer using a sterile 200-µL pipette tip. To remove any dislodged cells, the wells were rinsed twice with PBS before being filled with serum-free medium to minimize proliferation-related effects during the migration assay. Because CAL27 and SCC25 cells retain relatively strong epithelial characteristics and show moderate baseline migratory capacity, a 48-hour incubation period under serum-free conditions was selected to allow sufficient wound closure and facilitate reliable comparison between groups. Photomicrographs of the wound were captured with an inverted microscope immediately after the scratch was made (0 h) and again at the 48-hour mark. We quantified the extent of wound closure over time using ImageJ software. Wound closure was calculated based on the change in wound area relative to the initial wound area at 0 h.

### TIMER2.0 database analysis

To investigate the immunological landscape, this study employed TIMER2.0, an updated version of the TIMER web server that enables a thorough examination of tumor-infiltrating immune cells in over 30 different cancer types^35^. We utilized this platform to evaluate the correlation between TMEM92 and the presence of various immune populations within HNSCC, including B cells, CD4 + T cells, CD8 + T cells, neutrophils, macrophages, dendritic cells, and natural killer cells.

### Immune infiltration analysis by ssGSEA

To compare immune cell populations, we performed single-sample gene set enrichment analysis (ssGSEA), a computational method derived from the GSEA algorithm that is frequently used for immune profiling^36^. By implementing the GSVA R package, we generated enrichment scores for 28 distinct immune cell types. These scores were subsequently used to contrast the immune landscape between patient groups exhibiting high versus low expression of TMEM92.

### TISIDB database analysis

TISIDB (http://cis.hku.hk/TISIDB/index.php) is a web-based platform that enables users to explore the role of specific genes in tumor-immune interactions through literature mining and high-throughput data integration^37^. In this study, we utilized TISIDB to evaluate the relationship between TMEM92 and genes related to immunoinhibitors, immunostimulators, and the major histocompatibility complex (MHC) in HNSCC. Correlations with |R| > 0.2 and *p* < 0.05 were visualized.

### TIDE-based immunotherapy response prediction

To forecast the potential response to immune checkpoint inhibitors (ICIs), we utilized the Tumor Immune Dysfunction and Exclusion (TIDE) platform, a tool that has significantly enhanced comprehension of the tumor microenvironment (TME) by offering an efficient predictive model^38^. For this purpose, expression data from the TCGA-HNSCC dataset was uploaded to the TIDE portal to generate predictive scores. These TIDE scores were subsequently contrasted between the patient groups exhibiting high and low levels of TMEM92 expression.

### Statistical analysis

Several statistical methods were used to evaluate the clinical significance of TMEM92 in HNSCC. The diagnostic performance of TMEM92 expression was assessed using receiver operating characteristic (ROC) curve analysis. The association between TMEM92 expression and overall survival was evaluated using Kaplan–Meier survival analysis. To determine its independent prognostic value, univariate and multivariate Cox regression analyses were performed. Variables included in the multivariate model were selected based on clinical relevance and consideration of potential collinearity. Associations between TMEM92 expression and clinicopathological characteristics were analysed using the chi-square test, while Fisher’s exact test was used for clinical M because of the small sample size. Cases with Nx status were excluded from nodal status analysis.

All statistical analyses were performed using SPSS (v22.0), R software (v4.3.0), and GraphPad Prism (v10.1.2). Comparisons between two groups were conducted using Student’s t-test, whereas comparisons among multiple groups were performed using one-way ANOVA. A *P* value < 0.05 was considered statistically significant.

## Electronic Supplementary Material

Below is the link to the electronic supplementary material.

Supplementary Material 1

## Data Availability

The datasets analysed during the current study are publicly available in The Cancer Genome Atlas (TCGA) and Gene Expression Omnibus (GEO) repositories under accession numbers GSE184616, GSE181919, GSE103322, and GSE208253. Other data supporting the findings of this study are available from the corresponding author upon reasonable request.

## Abbreviations

TMEM92: Transmembrane protein 92
HNSCC: Head and neck squamous cell carcinoma
EMT: Epithelial–mesenchymal transition
TMEMs: Transmembrane proteins
TCGA: The cancer genome Atlas
GEO: Gene expression omnibus
GEPIA2: Gene expression profiling interactive analysis 2.0
UMAP: Uniform manifold approximation and projection
p-EMT: Partial EMT
TIMER2.0: Tumor immune estimation resource 2.0
IHC: Immunohistochemistry
CCK-8: Cell counting Kit-8
OS: Overall survival
KEGG: Kyoto Encyclopedia of genes and genomes
GO: Gene ontology
siRNA: Small interfering RNA
NC: Negative control
GSEA: Gene set enrichment analysis
ssGSEA: Single-sample gene set enrichment analysis
MHC: Major histocompatibility complex
TME: Tumor microenvironment
ICIs: Immune checkpoint inhibitors
ROC: Receiver-operating characteristic
AUC: Area under the ROC curve
BP: Biological processes
MF: Molecular functions
CC: Cellular components
MET: Mesenchymal-epithelial transition
Tregs: Regulatory T cells
Tfh: T follicular helper cells

## Declarations

### Declarations of generative AI and AI-assisted technologies in the writing process

During the preparation of this work, the authors used GPT-4o for grammar checking and language polishing. All authors have reviewed and approved the final version of the manuscript before submission.
