# Supplementary material for: TMEM92 drives EMT-associated invasiveness, cisplatin resistance, and immune suppression in head and neck squamous cell carcinoma
Source: Sci Rep. 2026 Apr 28;16:19498. doi: 10.1038/s41598-026-49694-7 (PMC13287742; doi:10.1038/s41598-026-49694-7)
Supplement: Supplementary file 1 — Supplementary Material 1 [file 41598_2026_49694_MOESM1_ESM.pdf]

Supplemental Figure 1 TMEM92 correlates with pathways related to cell migration and focal adhesion in HNSCC.

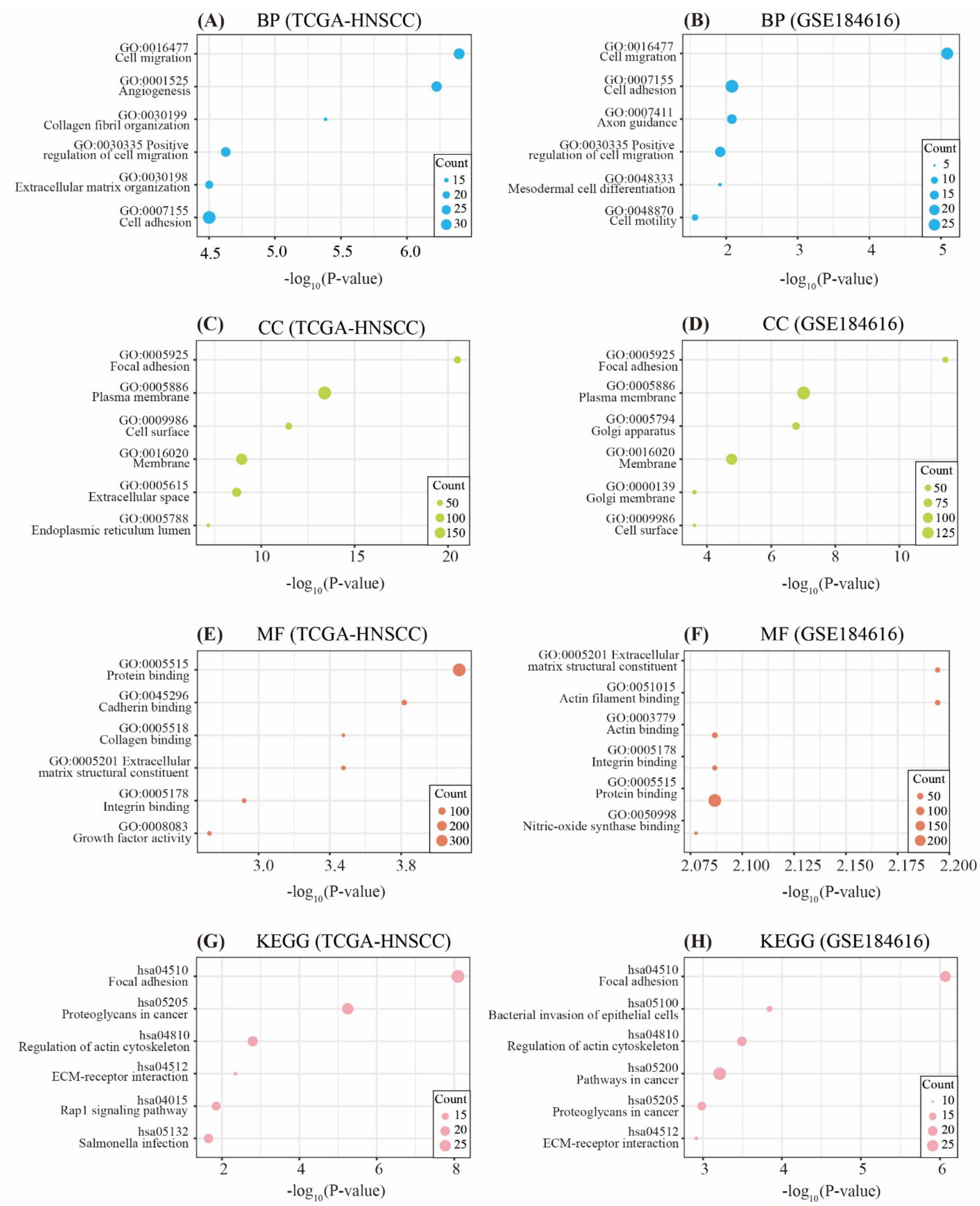

(A, C, E) Gene Ontology (GO) enrichment analysis of genes co-expressed with TMEM92 in The Cancer Genome Atlas (TCGA) database, displaying the most

significant terms for Biological Processes (BP), Cellular Components (CC), and Molecular Functions (MF). (G) Kyoto Encyclopedia of Genes and Genomes (KEGG) pathway analysis of TMEM92 in the TCGA database. (B, D, F) Corresponding GO enrichment analysis for genes co-expressed with TMEM92 in the GSE184616 dataset. (H) KEGG Pathway Enrichment Analysis for TMEM92-Associated Genes from the GSE184616 Dataset.

Supplemental Figure 2 Hallmark gene set enrichment analysis of TMEM92-associated pathways in HNSCC.

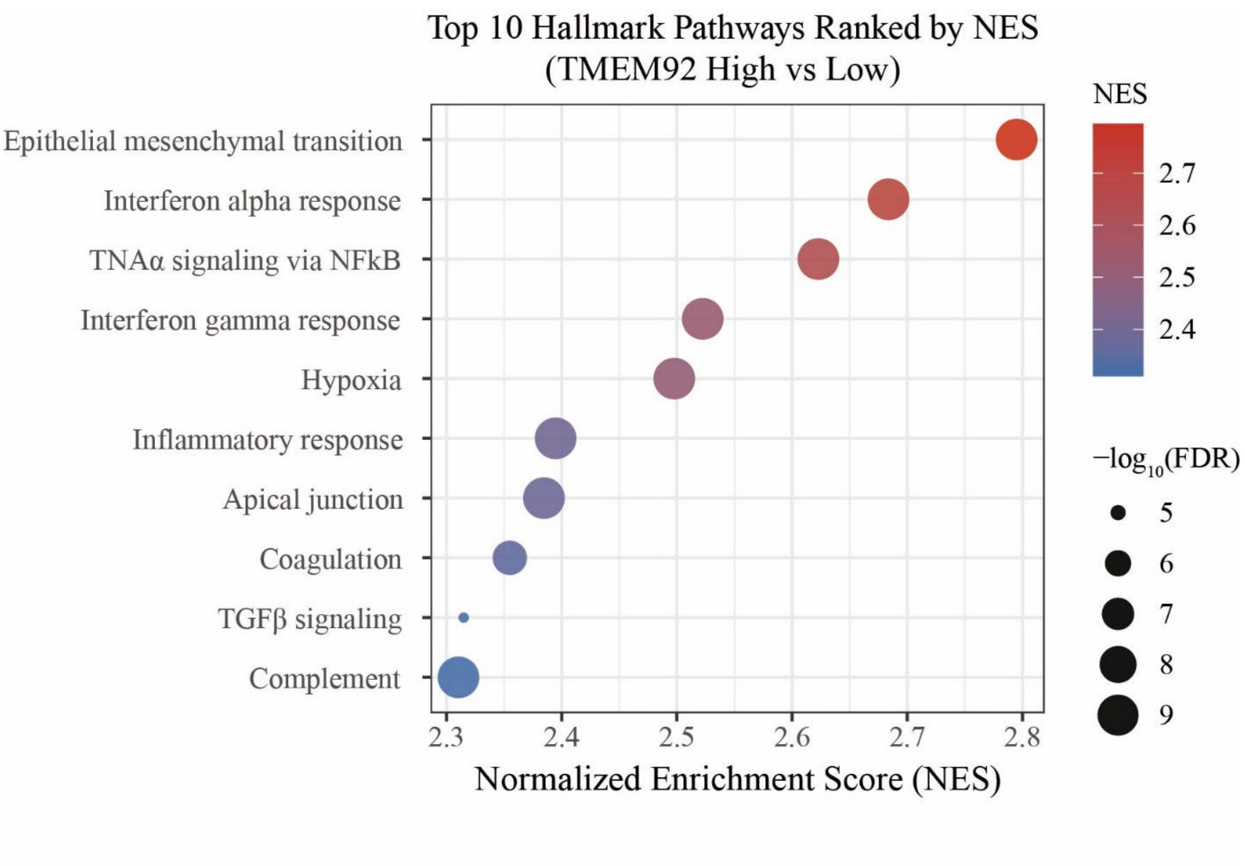

Bubble plot showing the top 10 enriched Hallmark pathways in the TMEM92-high group based on GSEA analysis.

Supplemental Figure 3. TMEM92 is significantly correlated with PTK2, TGFB1, and

NFKB1 in HNSCC.

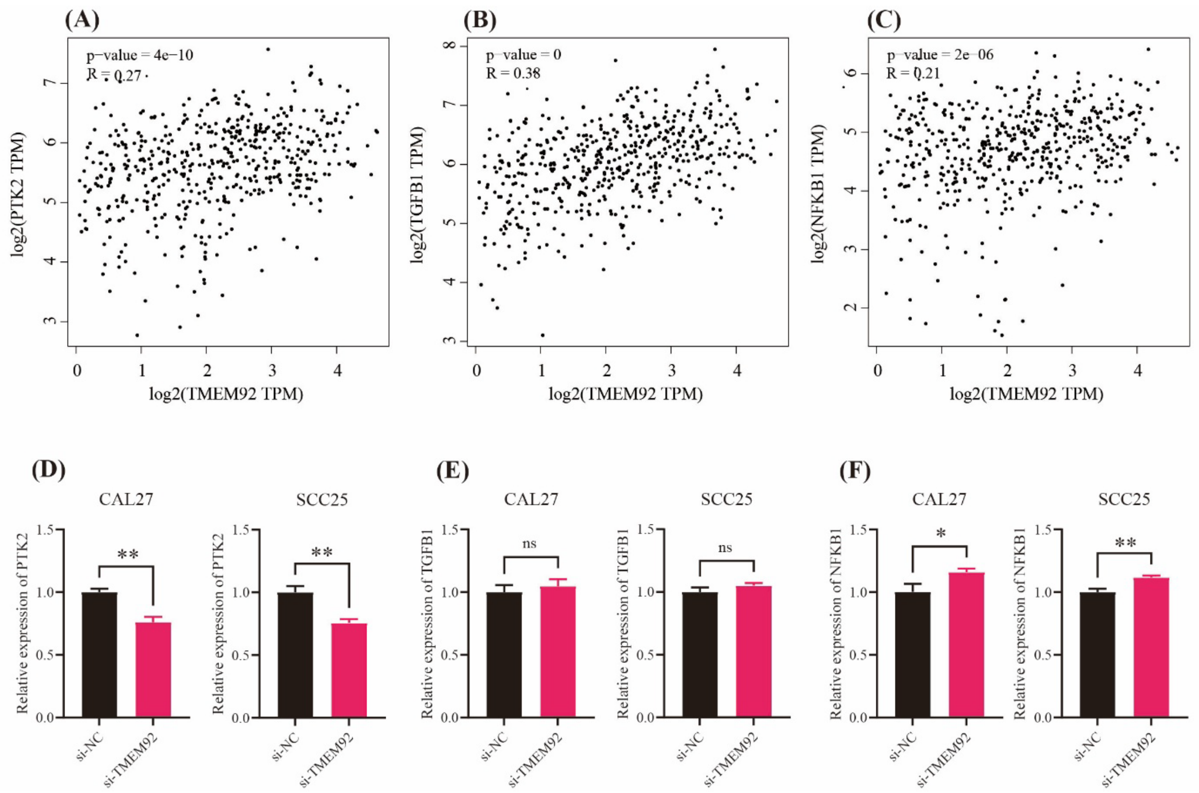

(A–C) GEPIA2.0 analysis showing significant positive correlations between TMEM92 and PTK2, TGFB1, and NFKB1 in HNSCC. (D–F) RT-qPCR analysis showing that TMEM92 knockdown decreased PTK2 expression, had no significant effect on TGFB1 expression, and increased NFKB1 expression. *P < 0.05; **P < 0.01; ***P < 0.001; ns, not significant.

Supplemental Figure 4 Dose–response curves and IC50 analysis of cisplatin in parental and resistant HNSCC cell lines after 48 h of treatment.

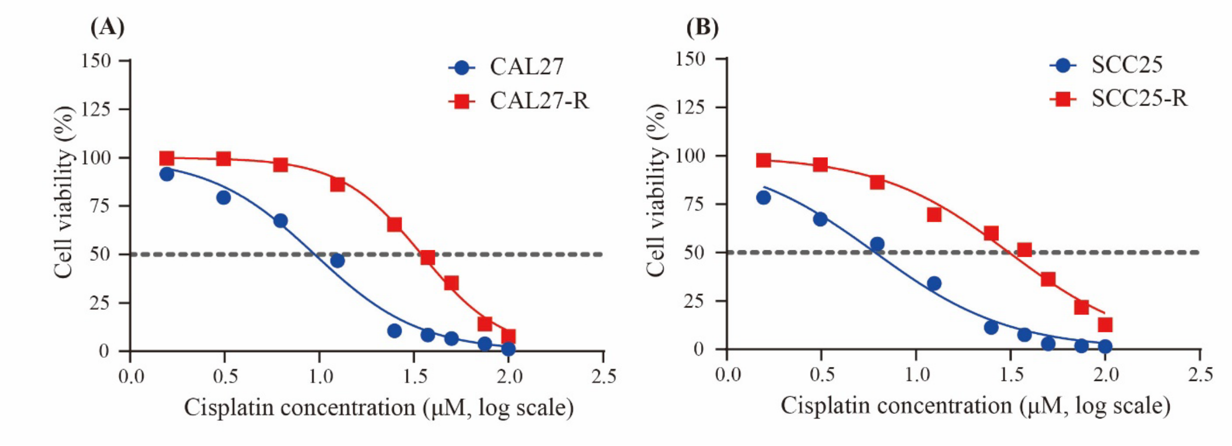

(A, B) Parental CAL27 and SCC25 cells and their corresponding resistant derivatives (CAL27-R and SCC25-R) were treated with increasing concentrations of cisplatin for 48 h. The resistant cell lines exhibited markedly higher IC50 values than their parental counterparts.

Supplemental Figure 5 Downregulation of TMEM92 enhances the sensitivity of HNSCC cells to cisplatin.

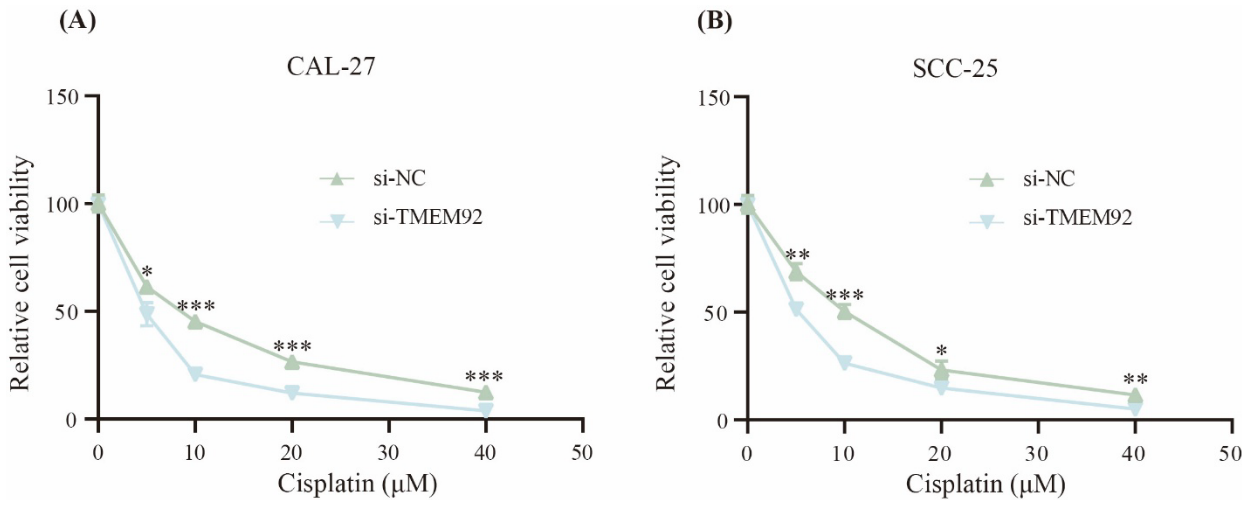

(A, B) CCK-8 experiment showed that cisplatin treatment impairs HNSCC cell viability, and si-TMEM92 treatment further impairs cell viability. *P < 0.05; **P <

0.01; ***P < 0.001; ns, not significant.

Supplemental Figure 6 Correlation between TMEM92 and immune infiltration in HNSCC based on TIMER2.0 online database and ssGSEA analysis.

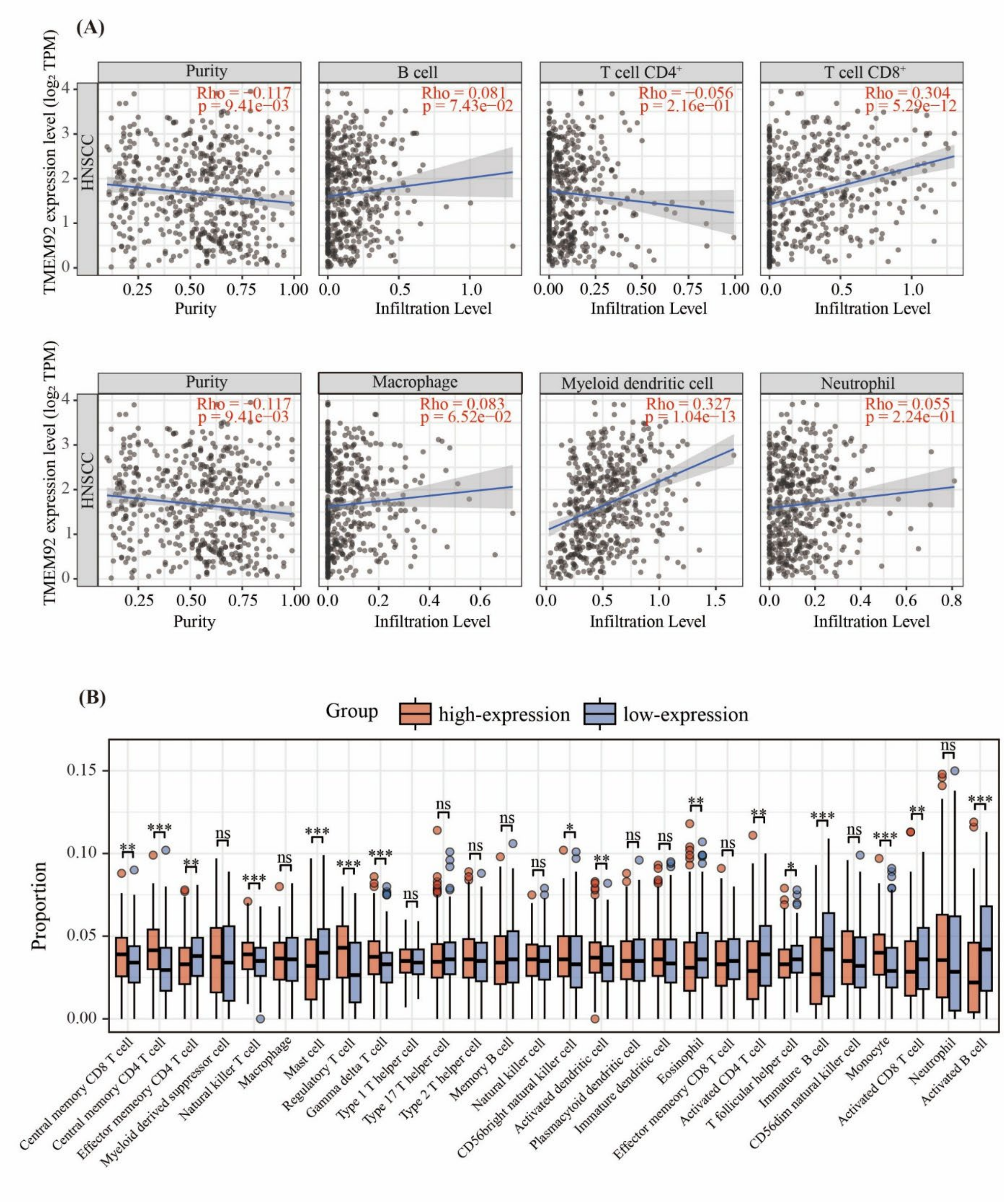

(A) Analysis of the correlation between TMEM92 expression and the infiltration levels

of various immune cell types in HNSCC. (B) Results from single-sample gene set enrichment analysis (ssGSEA) illustrating the relationship between TMEM92 expression and the enrichment scores of 28 distinct immune cell populations in HNSCC. *P < 0.05; **P < 0.01; ***P < 0.001; ns, not significant.

Supplemental Figure 7 Spatial transcriptomic analysis of the association between TMEM92-positive regions and T-cell distribution in HNSCC.

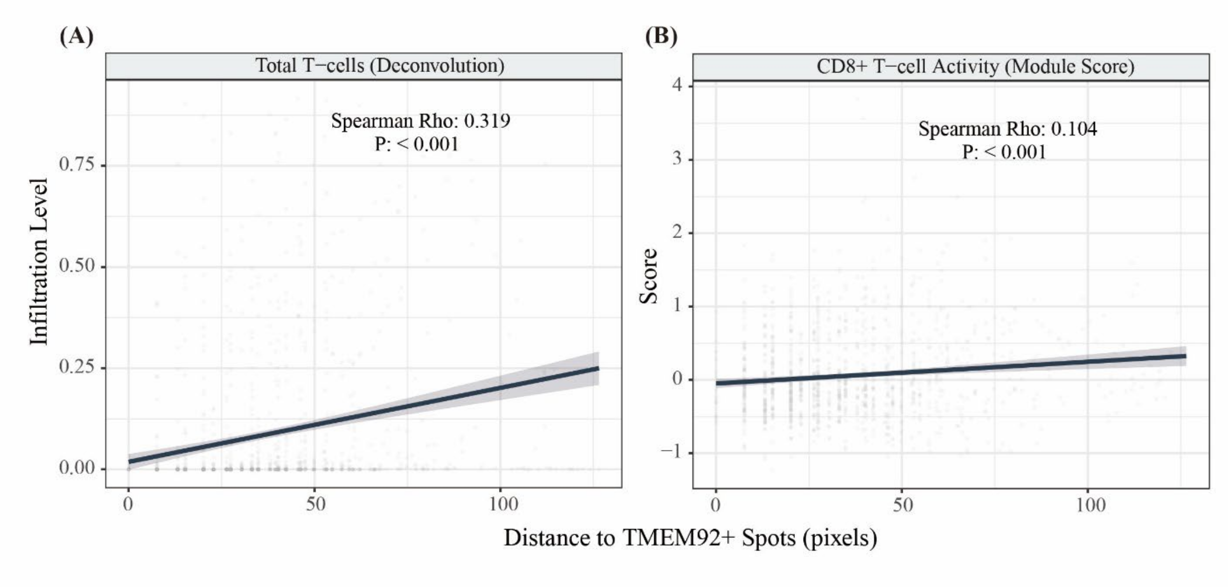

(A) Increasing distance from TMEM92-positive spots was significantly associated with higher overall T-cell abundance estimated by deconvolution in the GSE208253 dataset. (B) Increasing distance from TMEM92-positive spots was also positively correlated with $CD8^+$ cytotoxic T-cell activity assessed by module score.

Supplemental Figure 8 Correlation between TMEM92 and immune infiltration in HNSCC based on TISIDB online database and TIDE analysis.

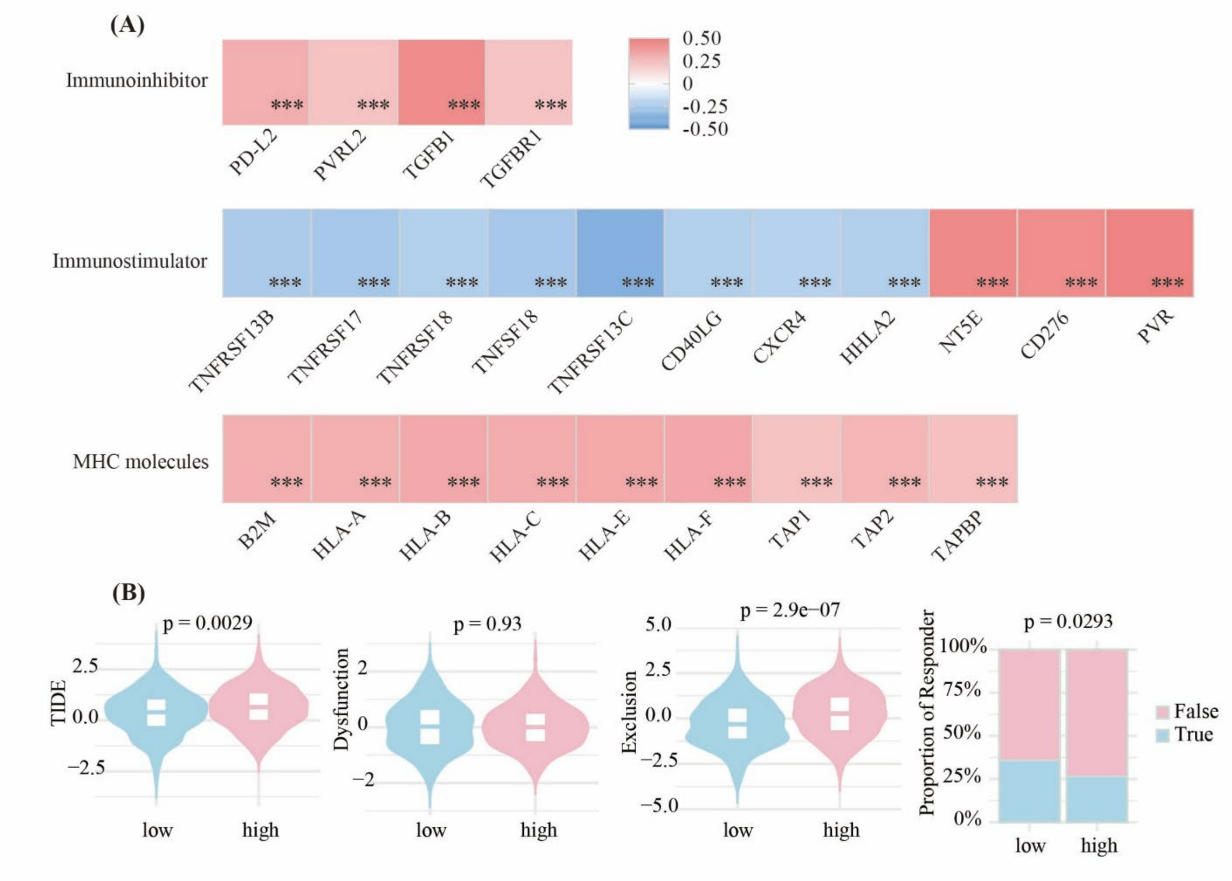

(A) Heatmap illustrating the correlation between TMEM92 and immunomodulators. (B) TIDE analysis showing that higher TMEM92 expression is associated with reduced predicted responsiveness to immunotherapy. *P < 0.05; **P < 0.01; ***P < 0.001; ns, not significant.

Supplemental table 1

clinical information and histopathologic characteristics of 10 cases

case_id	gender	age	site_of_resection	pathological_stage	clinical_TNM
1	male	55	Tongue	I	T3N0M0
2	female	71	Tongue	I-II	T3N0M0
3	male	53	Tongue	I-II	T3N2cM0
4	male	65	Tongue	I-II	T3N1M0
6	male	71	Tongue	I-II	T3N1M0
8	male	60	Tongue	I	T2N0M0
11	male	68	Tongue	I	T3N0M0
12	male	57	Cheek mucosa	I-II	T4aN1M0
14	female	61	Hard palate	I-II	T4bN0M0
17	male	65	Cheek mucosa	I-II	T4bN1M0

Original Images for Blots

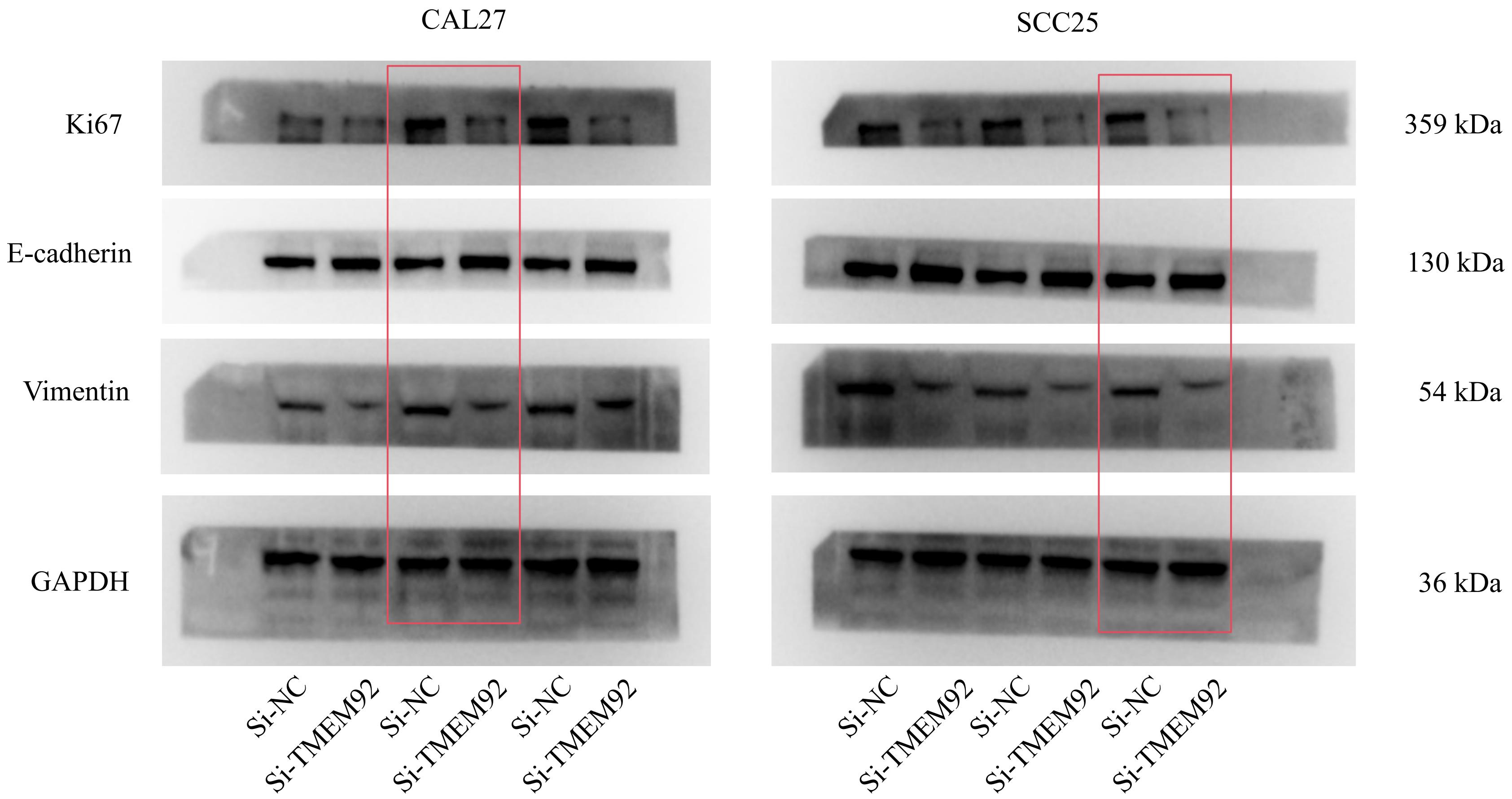

Representative original blots used for the final figures are highlighted within the red rectangles.
